# Cohesin Is Required for Activation of *MYC* by Estradiol

**DOI:** 10.1371/journal.pone.0049160

**Published:** 2012-11-08

**Authors:** Miranda V. McEwan, Michael R. Eccles, Julia A. Horsfield

**Affiliations:** Department of Pathology, Dunedin School of Medicine, University of Otago, Dunedin, New Zealand; Karolinska Institutet, Sweden

## Abstract

Cohesin is best known as a multi-subunit protein complex that holds together replicated sister chromatids from S phase until G2. Cohesin also has an important role in the regulation of gene expression. We previously demonstrated that the cohesin complex positively regulates expression of the oncogene *MYC*. Cell proliferation driven by MYC contributes to many cancers, including breast cancer. The *MYC* oncogene is estrogen-responsive and a transcriptional target of estrogen receptor alpha (ERα). Estrogen-induced cohesin binding sites coincide with ERα binding at the *MYC* locus, raising the possibility that cohesin and ERα combine actions to regulate *MYC* transcription. The objective of this study was to investigate a putative role for cohesin in estrogen induction of *MYC* expression. We found that siRNA-targeted depletion of a cohesin subunit, RAD21, decreased *MYC* expression in ER-positive (MCF7 and T47D) and ER-negative (MDA-MB-231) breast cancer cell lines. In addition, RAD21 depletion blocked estradiol-mediated activation of *MYC* in ER-positive cell lines, and decreased ERα binding to estrogen response elements (EREs) upstream of *MYC*, without affecting total ERα levels. Treatment of MCF7 cells with estradiol caused enrichment of RAD21 binding at upstream enhancers and at the P2 promoter of *MYC*. Enriched binding at all sites, except the P2 promoter, was dependent on ERα. Since RAD21 depletion did not affect transcription driven by an exogenous reporter construct containing a naked ERE, chromatin-based mechanisms are likely to be involved in cohesin-dependent *MYC* transcription. This study demonstrates that ERα activation of *MYC* can be modulated by cohesin. Together, these results demonstrate a novel role for cohesin in estrogen-mediated regulation of *MYC* and the first evidence that cohesin plays a role in ERα binding.

## Introduction

Over-expression of the *MYC* proto-oncogene is one of the most common oncogenic events in human cancers [1]. MYC is a pleiotropic transcription factor that has been found to bind to 10–15% of human genes [2]–[4]. MYC activation influences genes involved in multiple facets of tumor biology including proliferation [5]–[9], differentiation [10]–[14], apoptosis [15]–[18] and metastasis [19]–[23]. Recent studies demonstrate that MYC selectively binds to the promoter of active genes and amplifies their transcription [24], [25]. Rather than changing which genes are expressed, high levels of MYC increase the transcriptional output of tumor cells [24]. Given its ability to amplify transcription, *MYC* expression needs to be tightly regulated, and in fact both *MYC* mRNA and MYC protein have short half-lives, allowing rapid adjustment of MYC levels in response to various stimuli [26]. *MYC* is located in the human chromosome 8q24 region, a 2 MB segment of chromosome 8 that contains susceptibility loci for several diseases including colorectal, ovarian, thyroid, prostate and breast cancer [27]–[34].

*MYC* has a normal physiological role in mammary gland development [35], where it is a transcriptional target of the estrogen receptor (ER) and several other regulators [36], [37]. High levels of *MYC* have been observed in breast cancer cases, both at the mRNA (22–35%) and protein (41–45%) level [38]. A higher proportion of breast cancers over-express MYC at the protein or mRNA level than exhibit *MYC* amplification. Therefore, in the majority of breast cancers, over-expression of MYC is likely to be due to dysregulation of transcription, translation or protein stability [38]. In ER-positive breast cancer cells, estrogen stimulates *MYC* transcription, which in turn drives proliferation [37]. In ER-negative breast cancer, the genetic signature of hormone-driven proliferation can be reproduced in cancers that overexpress MYC [39]. This finding is consistent with the idea that MYC regulates a substantial number of the genes in the estrogen response pathway [40].

Previous work by our group and others has shown that *MYC* transcription is positively regulated by the protein complex, cohesin, in *Drosophila* [41], [42], zebrafish (*myca*) [42], mouse [43], [44] and human [45]. The Cohesin complex is composed of four core subunits: structural maintenance of chromosomes (SMC) subunits SMC1 and SMC3, and two non-SMC subunits, RAD21, and SCC3/Stromalin (SA) [46]. Cohesin is essential for proper chromosome segregation and DNA repair post-replication [46], [47] and has been shown to regulate gene expression [41], [45], [48]–[50], potentially by organizing higher-order chromatin conformation [51]–[53] or by interaction with chromatin remodeling complexes [54].

Over-expression, under-expression and mutations of cohesin subunits have been found in a variety of cancers, including breast cancer [55]. Single nucleotide polymorphisms (SNPs) have been identified in *RAD21* that predict susceptibility to breast cancer [56], [57]. In addition, clinical breast cancer samples have higher *RAD21* mRNA levels than normal breast tissue, and these higher levels are associated with poor prognosis [58]. Taken together these findings suggest that cohesin has potential to contribute to breast cancer pathology. Moreover, RAD21 depletion inhibited proliferation and sensitized breast cancer cell lines to Etoposide and Bleomycin, suggesting that targeting cohesin may be an effective treatment either alone or in combination with chemotherapy [57]. A small hairpin RNA (shRNA) screen aiming to identify genes that contribute to tamoxifen resistance in breast cancer cells found that depletion of several individual cohesin subunits increased sensitivity to tamoxifen [59], whereas an overexpression study found that high levels of RAD21 correlated with tamoxifen resistance [60]. A small interfering RNA (siRNA) screen to find druggable targets that are synthetic lethal in *MYC-*overexpressing cells identified *RAD21* [61]. Silencing of *RAD21* resulted in apoptosis and DNA damage in cells over-expressing *MYC* [61]. These findings highlight the potential for a functional role for RAD21/cohesin in MYC-driven breast cancer.

A genome-wide binding study revealed tissue-specific, inducible cohesin binding in breast and liver cancer cells [50]. In hormone-dependent MCF7 breast cancer cells, cohesin binds specific regions of the genome in response to estrogen [50]. Cohesin and ERα binding co-localize at regulatory regions in close proximity to genes that convert estrogen signals to cell growth and endocrine response [50]. Many of the regions where cohesin and ERα co-localize are involved in chromatin interactions that have potential to bring regulatory elements into proximity to gene promoters, which may facilitate transcription [62], [63]. Cohesin may contribute to estrogen-mediated regulation of responsive genes by stabilizing these chromatin loops [50], [62].

Analysis of global binding data revealed co-binding of ERα and cohesin at several regulatory elements within, and upstream of *MYC* [50]. In the present study, our aim was to investigate whether cohesin plays a role in estrogen-mediated activation of *MYC*. Consistent with our previous data, we found that cohesin depletion blocked *MYC* expression in breast cancer cell lines, and prevented its transcriptional induction by estrogen. We show that cohesin is necessary for ERα binding to specific sites within the 8q24 region, and hypothesize that cohesin modulation of ERα binding contributes to estrogen induction of *MYC*.

## Results

### Cohesin Maintains *MYC* Levels in Breast Cancer Cell Lines and is Required for Estradiol-induced Activation of *MYC*

**Figure 1:**
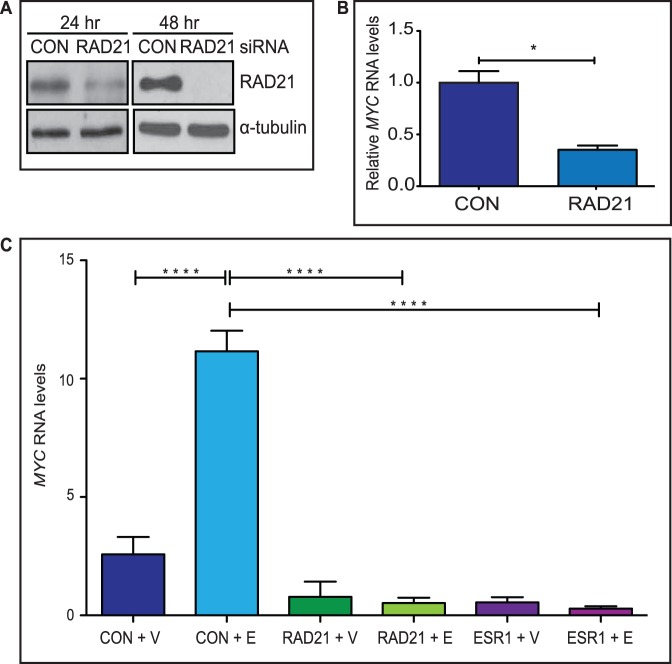
Depletion of RAD21 prevents estradiol activation of *MYC* in MCF7 cells. **A)** ***RAD21*** **silencing in MCF7 cells.** RAD21 protein levels were reduced 24 hours after transfection and completely depleted 48 hours after transfection with 10 nM RAD21 siRNA **B)** **RAD21 positively regulates** ***MYC*** **in MCF7** **cells**. Relative levels of *MYC* mRNA in Control (CON) and RAD21 siRNA transfected MCF7 cells were determined by qRT-PCR. Columns show the average values of relative normalized expression from three independent experiments. The * symbol indicates a significant (p<0.05) reduction in *MYC* transcript levels in RAD21 depleted MCF7 cells compared with cells transfected with Control siRNA. **C)** **Estradiol-activation of** ***MYC*** **is ERα- and RAD21-dependent**. MCF7 cells were transfected with Control (CON), RAD21 or ESR1 siRNA for 48 hours and then treated with vehicle (V) or estradiol (E) for 6 hours. *MYC* transcript levels are shown relative to Control siRNA + V treated cells. The results shown are the mean (+/− SEM) of 3 biological replicates. The **** symbol indicates a highly significant (p<0.001) reduction in *MYC* expression in RAD21 and ESR1 siRNA transfected cells treated with estradiol compared with Control siRNA transfected cells treated with estradiol.

It was previously shown that cohesin positively regulates *MYC* expression in zebrafish and *Drosophila* [42], [64], and similar results have been found in mouse [43], [44] and human [45]. To determine whether cohesin regulates *MYC* in human breast cancer cell lines we transfected MCF7 cells with siRNA targeting the RAD21 subunit of cohesin. By 24 hours post-transfection, there was a 64% reduction in RAD21 protein levels, and complete loss of RAD21 by 48 hours after treatment, relative to controls (Figure 1A). Quantitative RT-PCR (qPCR) analysis of *MYC* mRNA levels following RAD21 depletion for 48 hours indicated a significant (p  = 0.0319) reduction in *MYC* transcript levels by 65% (Figure 1B). These data suggest that in breast cancer cells *MYC* expression is dependent on cohesin.

The *MYC* gene is activated by estradiol [36], [37], [65] and MYC plays a crucial role downstream of ERα to regulate genes in the estrogen response pathway [40], [66]. To determine whether cohesin is required for estradiol-mediated activation of *MYC*, we transfected hormone and growth factor depleted MCF7 cells with control, RAD21 or ESR1 siRNA for 48 hours, prior to treatment with estradiol for 6 hours (Figure 1C). Consistent with previous observations [36], [37], estradiol induction increased *MYC* expression by 4.3 fold. In contrast, RAD21 depletion completely blocked estradiol induction of *MYC* expression (Fig. 1C). Ablation of *ESR1* expression also prevented expression of *MYC*, consistent with estradiol induction of *MYC* expression being ERα-dependent.

**Figure 2:**
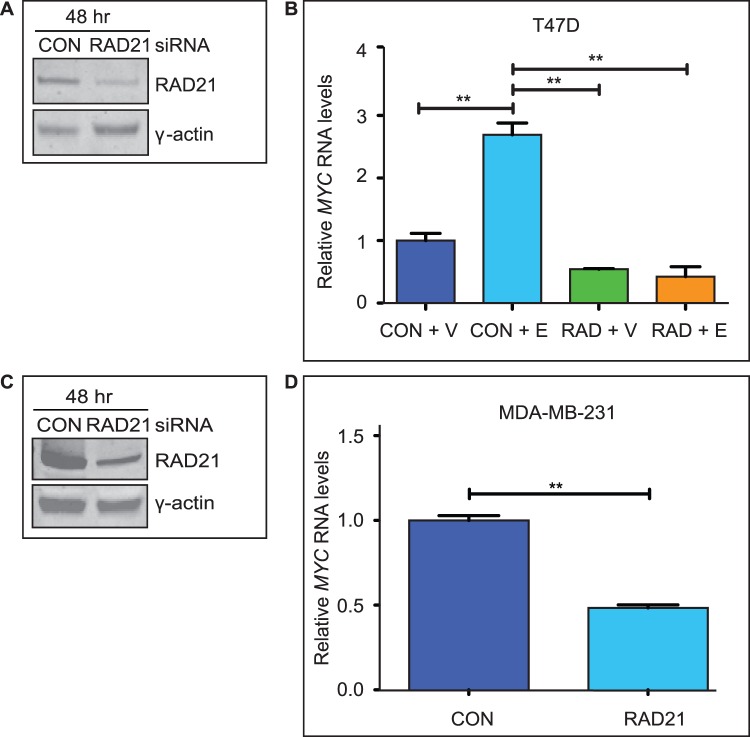
Depletion of RAD21 positively regulates *MYC* expression in both ER-positive and ER-negative breast cancer cells. **A)** ***RAD21*** **silencing in T47D cells.** RAD21 protein levels were reduced 48 hours after transfection with 10 nM RAD21 siRNA as described in B. **B)** **Estradiol-activation of** ***MYC*** **is RAD21-dependent in T47D cells**. T47D cells were transfected with Control (CON) or RAD21 (RAD) siRNA for 48 hours and then treated with vehicle (V) or estradiol (E) for 6 hours. *MYC* transcript levels are shown relative to Control siRNA + V treated cells. The ** symbols indicate a significant (p<0.01) reduction in *MYC* expression in RAD21-depleted cells relative to Control siRNA transfected cells, and between estradiol treated Control cells and estradiol treated RAD21-depleted T47D cells. **C)** **RAD21 silencing in MDA-MB-231 cells.** RAD21 protein levels were reduced 48 hours after siRNA transfection with 10 nM RAD21 siRNA. **D) RAD21 positively regulates** ***MYC*** **in ER-negative MDA-MB-231 cells.** Relative levels of *MYC* mRNA in Control (CON) and RAD21 siRNA transfected MDA-MB-231 cells were determined by qRT-PCR. All results are representative or the mean +/− SEM of three independent experiments. The symbol ** indicates a significant (p<0.01) difference in *MYC* transcript levels between Control siRNA and RAD21 siRNA transfected cells MDA-MB-231 cells.

To determine whether cohesin is required for estradiol-mediated activation of *MYC* in other ER-positive cell lines, we used siRNA to deplete RAD21 in T47D breast cancer cells. After 48 hours of transfection, there was a 50% reduction in RAD21 protein levels relative to control cells (Figure 2A). Similar to our observations in MCF7 cells, RAD21 depletion in T47D cells prevented activation of *MYC* expression by estradiol (Figure 2B).

High constitutive expression of *MYC* has been suggested to contribute to hormone-independence in ER-negative breast cancer cell lines [39], [66]–[69]. To determine whether *MYC* expression in ER-negative cell lines is also cohesin dependent, we transfected the ER-negative MDA-MB-231 cell line with RAD21 siRNA. This resulted in a 66% decrease in RAD21 protein after 48 hours, compared with control cells (Figure 2C). RAD21 depletion was accompanied by a ∼50% reduction in *MYC* transcript levels in MDA-MB-231 cells (p  = 0.0083), suggesting that cohesin ablation can also block growth regulatory pathways that converge to activate *MYC* expression in ER-negative breast cancer cell lines.

These results are consistent with our previous finding [42] that cohesin requirement for normal *MYC* transcription is conserved across multiple species. In addition, our data show that even a partial reduction of RAD21 completely blocks the transcriptional response of *MYC* to estradiol, or other growth regulatory pathways, in breast cancer cell lines.

### Analysis of Existing Data Reveals Overlapping Binding of ERα and Cohesin at Several *MYC* Regulatory Sites

**Figure 3:**
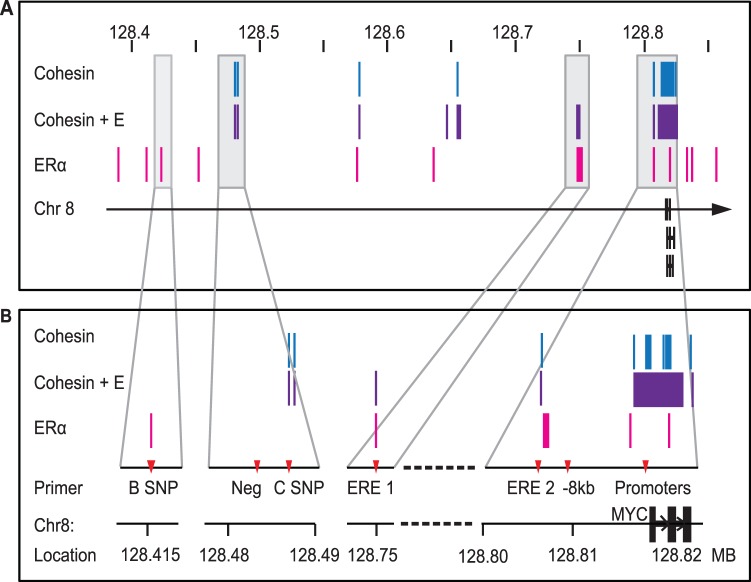
ER and cohesin binding overlap at several *MYC* regulatory sites. A) Schematic of locations of cohesin- and ER-binding within the 8q24 region. Genomic binding of cohesin and ERα were identified by ChIP-seq (Schmidt *et al*, 2010). Peaks of cohesin- (blue), estradiol-induced cohesin- (Cohesin + E, purple) and ERα-binding are indicated above the genome annotation of *MYC.* **B)** **Schematic of position of ChIP primers.** Specific regions of interest are magnified to indicate genomic binding identified by ChIP-seq and the location of primers used for Chromatin Immunoprecipitation (ChIP). Primer sequences are listed in Table S1. A scale diagram of primer positions relative to the *MYC* gene and promoters is shown in Figure S3.

**Figure 4:**
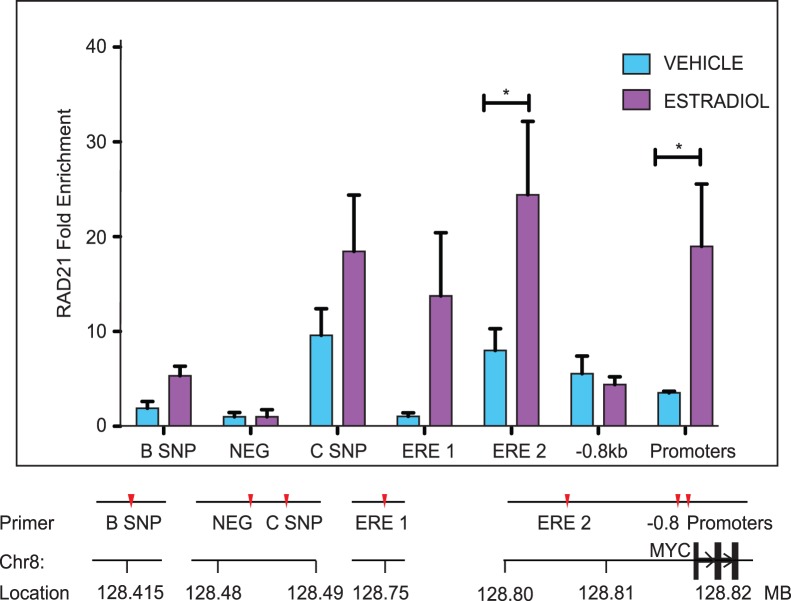
RAD21 binding is enriched by estradiol treatment at *MYC* regulatory sites within the 8q24 region. MCF7 cells were fixed following treatment with vehicle (V) or estradiol (E) for 45 minutes. RAD21 binding was analyzed by ChIP. Data are presented as fold enrichment relative to input chromatin and normalized against a negative site (NEG) where no binding was observed. The bar graph represents the mean +/− SEM of three independent experiments. The symbol * indicates a significant increase (p<0.05) in RAD21 binding between vehicle and estradiol treated cells. A schematic of primer locations is shown below the histogram. ChIP primer sequences are listed in Table S1. A scale diagram of primer positions relative to the *MYC* gene and promoters is shown in Figure S3.

Genome-wide binding analysis conducted by others demonstrated that estrogen-regulated genes are enriched for binding of both ERα and cohesin, and that cohesin binding to estrogen-regulated genes increases with estradiol treatment in MCF7 cells [50]. Cohesin binding is enriched at sites involved in ERα-anchored chromatin looping [50], [70]. Several binding sites for cohesin were found throughout the *MYC* locus and 8q24 region, a subset of which are induced by estradiol and co-localize with ERα-binding sites (Figure 3A). We designed Chromatin Immunoprecipitation (ChIP) primers to investigate RAD21- and ERα-binding at regions of interest (Figure 3B, Table S1, Figure S3). The ‘B SNP’ (393 kb upstream of *MYC* transcriptional start site) and ‘C SNP’ (−335 kb) ChIP amplicons correspond to genomic sites containing SNPs associated with cancer susceptibility. These sites are thought to be tissue-specific enhancers that interact with the *MYC* promoter [28], [71]–[75]. The ‘ERE 1′ site is an estrogen response element (ERE) 67 kb upstream of the *MYC* transcriptional start site and appears to be critical for estrogen responsiveness of *MYC* [76]. The ‘ERE 2′ site (−9.6 kb) was chosen because ERα and cohesin bind in close proximity in this region according to genome-wide binding data [50]; this site also displays insulator-like properties in several cell lines analyzed by the ENCODE project [77]. The ‘Promoters’ ChIP amplicon spans the P2 promoter of *MYC*. Finally, a site 339 kb upstream of the transcriptional start site (NEG) was used as a negative control to normalize background binding because there was no predicted or experimental RAD21- or ERα-binding at that site (Figure 4).

### RAD21 Binding is Enriched by Estradiol Treatment at Regulatory Elements within and Upstream of the *MYC* Locus

Since cohesin is required for estradiol-mediated activation of *MYC* (Figure 1C), it is possible that its location on chromosomes near ERα binding sites might facilitate estradiol induction of *MYC* transcription. Using anti-RAD21 chromatin immunoprecipitation (ChIP) of chromatin from MCF7 cells treated with 100 nM estradiol for 45 minutes, we observed elevated RAD21 binding at specific sites relative to controls (Figure 4). Estrogen-induced binding to predicted sites (Figure 3A) was consistent with previously identified genome-wide binding, although we also found enrichment of estrogen-induced cohesin binding at the B SNP site that had not been identified previously (Figure 3) [50]. Importantly, we observed enrichment of RAD21 binding at regulatory regions thought to modulate *MYC* transcription [28], [50], [71]–[75]. The presence of estrogen-induced cohesin binding at *MYC* regulatory regions is consistent with the notion that such binding could have functional consequences for *MYC* transcription. *MYC* expression is primarily driven by the P2 promoter in human breast cancer cell lines [78]. We observed enrichment of RNA Polymerase II (Pol II) binding at the promoter region following estradiol stimulation of MCF7 cells, and also in MDA-MB-231 cells grown in complete medium (Figure S1). Estradiol stimulation of MCF7 cells likewise resulted in a 6-fold enrichment of RAD21 binding at the promoter region (Figure 4). This finding suggests that cohesin binding, coincident with Pol II, could be part of a complex that regulates *MYC* transcription.

### RAD21 Binding to *MYC* Regulatory Elements Varies between Breast Cancer Cell Lines

**Figure 5:**
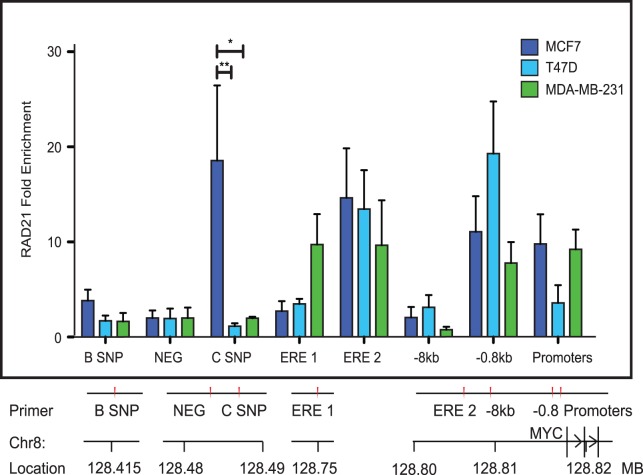
RAD21 binding to *MYC* regulatory elements varies between breast cancer cell lines. RAD21 binding in MCF7, T47D and MDA-MB-231 cell lines was analyzed by ChIP. RAD21 binding is shown as fold enrichment relative to input chromatin and normalized against a negative site (NEG) where no binding was observed. The bar graph represents the mean +/− SEM of three independent experiments. The symbols * and ** indicate significant differences (p<0.05 and p<0.01 respectively) in RAD21 binding relative to the MCF7 cell line. A schematic of primer locations is shown below the histogram. ChIP primer sequences are listed in Table S1. A scale diagram of primer positions relative to the *MYC* gene and promoters is shown in Figure S3.

*MYC* expression is constitutively higher in ER-negative breast tumors and hormone-independent breast cancer cell lines [39], [66]–[69]. *MYC* expression appears to correlate with *RAD21* expression [58], [79]. To determine whether RAD21 binding to regulatory elements could contribute to differences in *MYC* expression between breast cancer cell lines, we performed RAD21 ChIP on MCF7, T47D and MDA-MB-231 cells cultured in their respective complete media (Figure 5). MCF7 cells have high levels of RAD21 binding at the C SNP enhancer, compared with T47D (p<0.01) and MDA-MB-231 (p<0.05) cells which have no RAD21 binding at this site. There was 3-fold higher RAD21 binding at the ERE 1 site in MDA-MB-231 cells, although the difference is not significant. In summary, we found that there are slight differences in RAD21 binding between breast cancer cell lines, however more work needs to be done to determine whether the variability in binding at these sites is functionally significant.

### RAD21 is Necessary for ERα Binding within the *MYC* Locus, but cannot Influence Activity of an Exogenous Estrogen Response Element (ERE)

**Figure 6:**
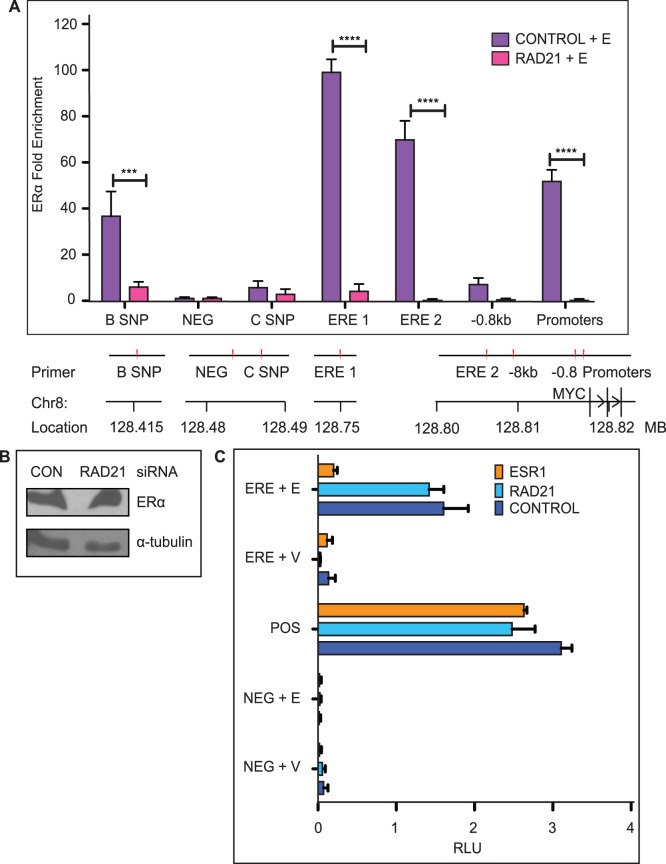
RAD21 is necessary for ERα binding within the *MYC* locus but not an exogenous ERE. A) RAD21 silencing abrogates ERα binding upstream of *MYC*. MCF7 cells were transfected with Control or RAD21 siRNA for 48 hours and treated with estradiol for 45 minutes before fixation. ERα binding was analyzed by ChIP. Data shown is fold enrichment; binding was calculated relative to input chromatin and normalized against the negative control site (NEG) where no binding is observed. The bar graph shows the mean +/− SEM of three independent experiments. The marks *** and **** indicate a highly significant (p<0.005 and p<0.001 respectively) decrease in ERα-binding in RAD21 siRNA transfected cells relative to Control siRNA transfected cells. A schematic of primer locations is shown below the histogram. ChIP primer sequences are listed in Table S1. A scale diagram of primer positions relative to the *MYC* gene and promoters is shown in Figure S3. **B)** **Total ERα levels are not affected by RAD21 depletion.** ERα levels remained unchanged 48 hours after transfection with 10 nM RAD21 siRNA. **C)** **RAD21 depletion does not affect ERE-mediated transcription of luciferase in a plasmid-based reporter system**. MCF7 cells were transfected for 48 hours with Control, RAD21 or ESR1 siRNA and either a negative control (NEG) plasmid, a positive control plasmid (POS) or a plasmid encoding the firefly luciferase reporter gene under the control of 5 EREs. Cells were treated with vehicle (V) or estradiol (E) for 24 hours before luciferase activity was measured. Firefly luciferase activity was normalized to *Renilla* luciferase activity, and expressed as relative luciferase units (RLU). The mean ± SEM (n  = 5 biological replicates) is shown. There was no significant difference in estradiol activation of ERE-luciferase between Control and RAD21-depleted cells.

**Figure 7:**
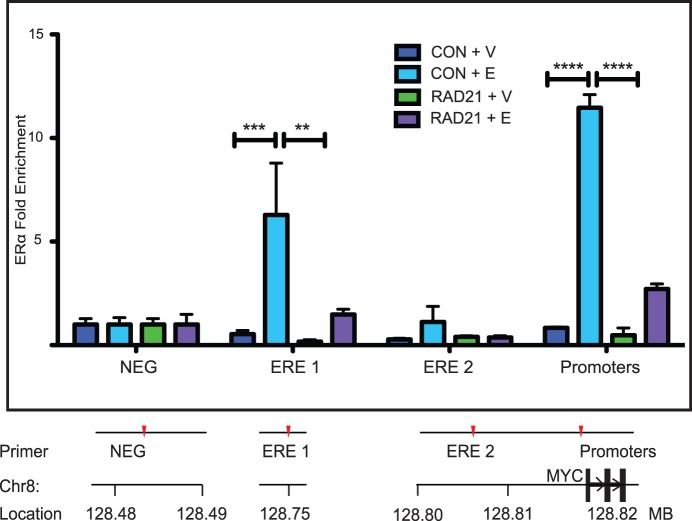
RAD21 depletion reduces ERα binding upstream of *MYC*. T47D cells were transfected with Control or RAD21 siRNA for 48 hours and treated with estradiol for 45 minutes before fixation. ERα binding was analyzed by ChIP. Data shown is fold enrichment; binding was calculated relative to input chromatin and normalized against the negative control site (NEG) where no binding is observed. The bar graph shows the mean +/− SEM of three independent experiments. The marks **, *** and **** indicate a highly significant (p<0.01, p<0.005 and p<0.001 respectively) decrease in ERα-binding in RAD21 siRNA transfected cells relative to Control siRNA transfected cells. A schematic of primer locations is shown below the histogram. ChIP primer sequences are listed in Table S1. A scale diagram of primer positions relative to the *MYC* gene and promoters is shown in Figure S3.

Consistent with existing data [36], [37], our results indicate that ERα is required for activation of *MYC* by estradiol (Figure 1C). A previous study showed that a single enhancer 67 kb upstream of the *MYC* transcriptional start site (ERE 1) is competent to confer estrogen responsiveness to the *MYC* promoter [76]. To determine if cohesin facilitates binding of ERα to this enhancer, or to other regulatory elements within the 8q24 region, we performed ChIP using an antibody detecting ERα in RAD21-depleted, estradiol-treated MCF7 and T47D cells (Figure 6A and 7). We observed a highly significant (p<0.001) reduction of ERα binding at all sites examined in RAD21-depleted MCF7 cells (Figure 6A), and a similar decrease in ERα binding at ERE 1 and the promoter region in T47D cells (Figure 7). In contrast to the MCF7 cells we did not observe ERα binding at ERE 2 in T47D cells (Figure 7). The reduction in ERα binding was not due to a decrease in ERα, as ERα protein levels were unaffected by RAD21 knock down (Figure 6B and data not shown). To confirm that siRNA RAD21*-*depletion results in loss of chromatin-bound RAD21, we performed anti-RAD21 ChIP in RAD21-depleted, estradiol treated MCF7 cells (Figure S2). These data suggest that RAD21/cohesin binding facilitates ERα binding to certain sites within the *MYC* locus by an as yet unknown mechanism.

It is unlikely, but possible, that cohesin could somehow recognize the sequence of an ERE independently of ERα and chromatin context to effect a transcriptional response. To investigate whether cohesin can influence the transcriptional response of a naked ERE, we measured the effects of RAD21 or ERα depletion on the output of a transiently transfected ERE-luciferase reporter system in MCF7 cells (Figure 6C). Cells were treated with estradiol (E) or vehicle (V) for 24 hours before measurement of luciferase activity. Estradiol treatment elicited a ∼12 fold increase in luciferase activity in both control and RAD21-depleted cells. In contrast, ERα depletion completely blocked luciferase expression. The results indicate that cohesin cannot directly influence transcription from a naked ERE independent of the chromatin context.

### ERα is Necessary for Estradiol-induced Enrichment of RAD21 Binding within the *MYC* Region

**Figure 8:**
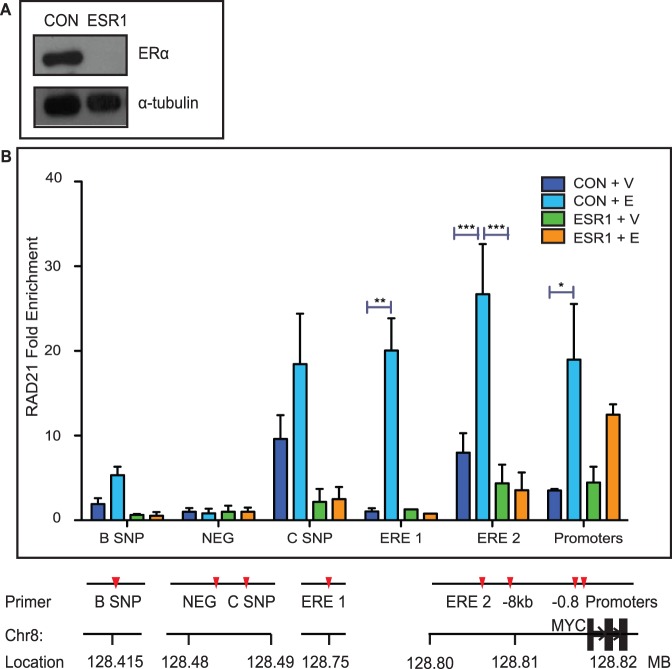
ERα is necessary for estradiol-mediated induction of RAD21 binding within the *MYC* locus in MCF7 breast cancer cells. A) *ESR1* silencing in MCF7 cells. ERα levels were depleted 48 hours after ESR1 siRNA transfection (10 nM). **B) ERα depletion prevents enrichment of RAD21 binding in response to estradiol.** MCF7 cells were transfected with Control or ESR1 siRNA for 48 hours and treated with estradiol (E) or vehicle (V) for 45 minutes before being fixed. RAD21 binding was analyzed using ChIP. Data shown is fold enrichment; binding was calculated relative input chromatin and normalized against the negative control site (NEG) where no binding was observed. The bar graph shows the mean +/− SEM of three independent experiments. The symbols *, ** and *** indicate significant (p<0.05, p<0.01 and p<0.005, respectively) enrichment in RAD21 binding in Control siRNA transfected cells treated with estradiol. There was no significant difference in RAD21 binding between ESR1 siRNA transfected cells treated with vehicle or with estradiol. ChIP primer sequences are listed in Table S1. A scale diagram of primer positions relative to the *MYC* gene and promoters is shown in Figure S3.

**Figure 9:**
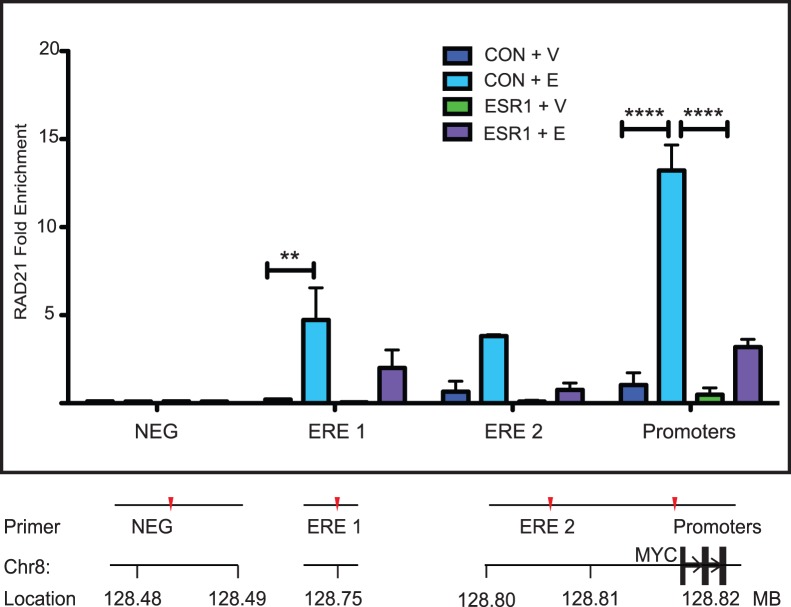
ERα depletion prevents enrichment of RAD21 binding in response to estradiol. T47D cells were transfected with Control or ESR1 siRNA for 48 hours and treated with estradiol (E) or vehicle (V) for 45 minutes before being fixed. RAD21 binding was analyzed using ChIP. Data shown is fold enrichment; binding was calculated relative input chromatin and normalized against the negative control site (NEG) where no binding was observed. The bar graph shows the mean +/− SEM of three independent experiments. The symbols ** and **** indicate significant (p<0.01 and p<0.0001, respectively) enrichment in RAD21 binding in Control siRNA transfected cells treated with estradiol. There was no significant difference in RAD21 binding between ESR1 siRNA transfected cells treated with vehicle or with estradiol. ChIP primer sequences are listed in Table S1. A scale diagram of primer positions relative to the *MYC* gene and promoters is shown in Figure S3.

Cohesin binding to the *MYC* region is enhanced by estradiol treatment ( [50], Figure 3A and B, Figure 4). Treatment of MCF7 cells with estradiol results in enriched RAD21 binding at specific sites within the *MYC* locus, including sites where ERα also binds (Figure 3A and B, Figure 4). Since the locations of ERα and cohesin binding frequently coincide, we were interested to determine whether RAD21 binding is influenced by ERα. Transfection of MCF7 or T47D cells with an *ESR1*-targeted siRNA markedly reduced levels of *ESR1* mRNA and ERα protein by 48 hours (Figure 8A and data not shown) without affecting constitutive levels of RAD21 binding in vehicle treated cells (Figure 8C and 9). ERα depletion prevented estradiol-induced enrichment of RAD21 binding at all sites tested except at the promoter region in MCF7 cells. In contrast, although estradiol-induced enrichment of RAD21 was reduced in the absence of ERα, this was primarily significant at the promoter region in T47D cells (p<0.001). The data suggest that ERα may facilitate RAD21 binding to regulatory elements upstream of *MYC* but that additional factors are likely to play a role.

## Discussion

### Regulation of *MYC* Transcription by Cohesin is Conserved in Multiple Breast Cancer Cell Lines

Because regulation of *MYC* by cohesin is conserved in several species [41]–[45], and *MYC* regulation is thought to be central to mechanisms of tumorigenesis in several types of cancer [27]–[34], it was of considerable interest to determine whether cohesin-mediated regulation of *MYC* is relevant to human cancers. We found that cohesin was indeed necessary to maintain *MYC* transcript levels in three human breast cancer cell lines (Figures 1B, C; 2B, D).

A study showing that RAD21 depletion prevents estrogen-induced G_0_/G_1_-S transition in breast cancer cells [50] is consistent with regulation of *MYC* by cohesin, since a large proportion of genes involved in estrogen-stimulated cell cycle progression are downstream of *MYC* [40]. Indeed, we determined that RAD21 depletion in ER-positive breast cancer cell lines prevented activation of *MYC* by estradiol (Figures 1C; 2B). RAD21 depletion had a small effect on constitutive levels of *MYC* expression in growth factor deprived, unstimulated cells. However, RAD21 depletion had a greater effect on *MYC* transcription in ER negative (MDA-MB-231) and ER positive (MCF7, T47D) cell lines undergoing rapid proliferation in complete medium containing steroids and growth factors (Figures 1B; 2D; and data not shown). These data indicate that cohesin is able to influence the input of multiple growth factor pathways into *MYC* expression, including hormone dependent regulation of *MYC*.

### RAD21 Binding Indicates Potential for Cohesin to Directly Influence Expression of Estrogen-regulated Genes in Breast Cancer

A genome-wide analysis in MCF7 cells revealed that cohesin binding co-localizes with ERα proximal to estrogen-regulated genes [50], raising the possibility that cohesin could directly contribute to regulation of these genes.

Several genomic locations upstream of the *MYC* locus at 8q24 recruit cohesin in response to estrogen (Figure 3). Some of these locations are in proximity to previously identified enhancers [28], [44], [50], [71]–[75], [80]. We confirmed the whole genome data by showing that RAD21 binds to regulatory elements within the 8q24 region, including enhancers and the *MYC* promoters (Figure 4). Interestingly, the sites with the greatest enrichment in RAD21 binding in response to estradiol are sites where ERα also binds.

A site 393 kb upstream of *MYC* (B SNP) is within a region associated with breast cancer susceptibility (rs13281615) [81] and was shown to interact long-range with the *MYC* promoter in MCF7 cells [28], however the polymorphism is not associated with differences in *MYC* expression in breast tumors [82]. We observed a small increase in RAD21 binding in response to estradiol at this site in MCF7 cells.

We confirmed that in MCF7 cells RAD21 binds a putative enhancer for *MYC* that we termed C SNP, due to its proximity to a SNP associated with colorectal (CRC) and prostate cancer [27], [29], [80], [83]–[89]. Several transcription factors, including TCF4, GFI and CDX2, also bind at this site and may influence *MYC* expression through long-range enhancer to promoter looping [72]–[74], [80], [90]–[93]. However, although a loop between the SNP and the *MYC* promoter was identified in MCF7 breast cancer cells [74], this loop is not anchored by either ERα [62] or Pol II [63], suggesting it does not mediate *MYC* transcription in this cell line.

A site 67 kb upstream of *MYC* containing a half ERE and AP1 binding site acts as an enhancer, conferring estrogen responsiveness to the *MYC* promoter [76]. ERα and cohesin bind this site (ERE 1) in estrogen-stimulated MCF7 and T47D cells [50], and we also found that RAD21 binds only when MCF7 or T47D cells are treated with estradiol. Significantly, the ERE 1 enhancer interacts with the *MYC* gene via a Pol II-anchored loop [63]. Therefore it is possible that this enhancer could be involved in cohesin regulation of *MYC* transcription.

We identified ERE 2 as a *MYC* proximal site with concurrent binding of ERα and cohesin from existing ChIP-seq data in MCF7 cells [50]. Integration of ChIP-Seq data using a Hidden Markov Model (HMM) suggests that this site is an insulator in 8/9 of the cell lines analyzed by the ENCODE consortium [77]. We confirmed that RAD21 binds to this site in unstimulated MCF7 but not T47D cells, and with 3-fold enrichment following estradiol stimulation in both cell lines, suggesting that this site may also play a role in regulating transcription of *MYC*.

### Differential Cohesin Binding at Selected Regulatory Elements in Breast Cancer Cell Lines

While RAD21 binding was consistent at some locations near *MYC* in all three breast cancer cell lines examined, there were differences at particular regulatory elements (Figure 5). For example, enrichment of RAD21 binding was observed at the C SNP enhancer in MCF7, whereas no binding was observed at this site in T47D or MDA-MB-231 cells. This site binds several different transcription factors and has been shown to interact with the *MYC* promoter in various cancer cell lines including MCF7 cells (the interaction has not been investigated in T47D or MDA-MB-231 cells to our knowledge) [72]–[74], [90]–[93]. It is unclear why RAD21 binding is enriched in MCF7 but not the other breast cancer cells, but it is possible that interaction between the C SNP enhancer and the *MYC* promoter contributes to regulation of *MYC* in MCF7 cells. Interestingly, we observed ∼3-fold higher RAD21 binding at the ERE 1 site in the ER-negative MDA-MB-231 cell line than the ER-positive MCF7 and T47D cell lines, although this was not statistically significant. It is possible that constitutive cohesin enrichment at this enhancer in ER negative cells mimics the effects of estradiol enrichment in the ER positive cells, contributing to hormone-independent activation of *MYC* transcription.

### Cohesin is Required for Binding of ERα to Regulatory Regions Upstream of the *MYC* Locus

Cohesin and ERα co-bind at estrogen-regulated genes [50] and we found that RAD21 binds to the promoter region of the *MYC* gene, together with several upstream regulatory elements. RAD21 depletion resulted in a loss of ERα-binding at all the locations we examined at the *MYC* locus and wider 8q24 region in MCF7 cells (Figure 6A) and a reduction in binding at estrogen responsive sites in T47D cells (Figure 7). Furthermore, reduced binding was not due to depletion of ERα protein (Figure 6B). Although a recently published paper suggests that cohesin depletion down-regulates ESR1 [94], our results (Figure 6B) are consistent with previous findings that knockdown of cohesin does not influence levels of hormone receptors in vertebrates [45], [95]. Our finding that RAD21 depletion did not alter ERE-driven luciferase expression in a reporter system (Figure 6C) argues against the idea that reduced ERα levels account for the results presented here.

As discussed previously, the ERE 1 enhancer confers estrogen responsiveness to the *MYC* promoter [76]. Importantly, RAD21 is necessary for a significant increase in ERα binding at this site in MCF7 or T47D cells. Genome-wide analysis of chromatin interactions associated with Pol II revealed an interaction in MCF7 cells between the ERE 1 and the *MYC* gene that may be important for transcription [63]. Cohesin is known to contribute to cell-type specific chromatin interactions, between enhancers and promoters, associated with gene regulation [49], [96], [97]. Cohesin may regulate *MYC* by stabilizing the loop between ERE 1 and the *MYC* gene.

### Potential Mechanisms for Cohesin-dependent Regulation of ERα Binding to *MYC* and cis-regulatory Elements

Since RAD21 depletion did not alter ERα protein levels (Figure 6B), there must be an alternative mechanism by which it modulates ERα binding. This mechanism cannot involve direct recognition of an ERE by cohesin, since RAD21 depletion had no effect on transcription from an exogenous ‘naked’ ERE reporter construct (Figure 6C). Possible mechanisms for the role of cohesin include the recruitment or modulation of other chromatin proteins that may cooperatively facilitate *MYC* transcription. In support of this idea, RAD21 was identified in a screen to identify epigenetic factors in human cells [98] and was found to directly interact with the ATPase SNF2h component of the ISWI chromatin remodeling complex [54]. Cohesin has previously been found to interact with the transcriptional co-activator Mediator to co-ordinate gene expression by loops between enhancers and promoters [49]. Interestingly, Mediator has also been shown to facilitate ERα binding to chromatin, but not to naked DNA [99], suggesting that cohesin and Mediator may act together in estrogen-induced gene expression. A recent study identified the histone demethylase JMJD2B as a co-factor for ERα in human breast cancer cells [100]. Demethylation of the histone H3K9Me3 is necessary for ERα to bind to chromatin, and JMJD2B facilitates estradiol-mediated induction of several genes including *MYC* [100]. Depletion of JMJD2B reduced *MYC* estrogen-responsiveness by about half in T47D cells [100]. It is possible that cohesin acts in combination with these other chromatin modifiers to allow access of ERα to the *MYC* locus in response to estrogen.

### Co-dependence of ERα and RAD21 Binding in the Regulation of *MYC* Transcription

Activation of *MYC* transcription in response to estradiol is accompanied by enrichment of RAD21 and ERα binding to *MYC* enhancers and the promoter region. Interestingly, we found that ERα depletion blocked estradiol-induced enrichment of RAD21 binding to the ERE 1 and ERE 2 regulatory elements, but not the promoter region in MCF7 cells (Figure 8B). In contrast in T47D cells (Figure 9), although RAD21 binding is highly enriched at ERE 1 and ERE 2 in response to estradiol (23-fold and 5-fold respectively), the most significant enrichment is at the promoter region (13-fold, p<0.0001). However, consistent with the MCF7 cells, there is no significant estradiol-induced enrichment of RAD21 binding in ERα-depleted T47D cells. Depletion of ERα did not significantly affect RAD21 binding to these sites in un-stimulated MCF7 or T47D cells (Figure 8B), implying a role for ligand-bound ERα in facilitating cohesin binding in response to estradiol.

Ligand-bound ERα recruits the SWI-SNF chromatin remodeling complex, thereby opening chromatin conformation to facilitate gene transcription [101]. It is possible that ER-induced chromatin modifications facilitate cohesin binding, or even locate cohesin to specific sites such as the ERE 1 enhancer. In contrast, we observed estradiol-induced enrichment of RAD21 binding to the promoter region even in the absence of ERα in MCF7 cells (Fig 8B). This suggests that the chromatin in this region is likely to be accessible without further remodeling. Promoter-proximal pausing of Pol II is an important mechanism of transcriptional regulation of *MYC* [102]. Interestingly, cohesin has been found to regulate transition of paused polymerase to elongation in *Drosophila* [103], and this role, if conserved, may account for its estrogen-independent presence at the *MYC* promoters.

### Conclusion

Taken together, our data suggest that cohesin and ERα may cooperatively and co-dependently alter chromatin context to activate *MYC* transcription in response to estradiol and other growth factor signaling pathways. The hypothesis that cohesin influences transcriptional response to estrogen via chromatin remodeling mechanisms warrants further investigation.

Our work has potential clinical implications for the treatment of breast cancer and other *MYC-*driven cancers. *RAD21* was included in the original “druggable genome” identified by Hopkins and Groom in 2002 [104]. Cohesin plays a role in tamoxifen- [60], [105], [106] and chemotherapy-resistance [57], [107], and its down-regulation induces radiation sensitivity [108]. A screen to identify “druggable genes” necessary for the survival of *MYC* over-expressing cells showed that down-regulation of RAD21 resulted in apoptosis and DNA damage [61]. Together with our results, these studies argue that targeting cohesin could have therapeutic potential for breast and other cancers.

## Methods

### Cells and Transfection

All culture reagents were from Invitrogen and Chemicals from Sigma unless otherwise indicated. (T47D). Human breast cancer cell lines (ERα-positive MCF7 [ATCC HTB-22]; ERα-positive T47D [ATCC HTB-133] and ERα-negative MDA-MB-231 [ATCC HTB-26]) were generously provided by Edwin Cheung (Genome Institute, Singapore) and Antony Braithwaite (University of Otago, Dunedin, New Zealand). Low passage number cells (p9– p15) were incubated in a 37°C humidified incubator at 5% CO_2_ (MCF7 and MDA-MB-231) or 10% CO_2_ (T47D). MCF7 and MDA-MB-231 cells were routinely cultured in DMEM supplemented with 10% FBS. T47D cells were cultivated in RPMI medium with 10% FBS. Cells were reverse transfected with 10 nM siRNA for 24 or 48 hours with RNAiMAX (Invitrogen). Cells were seeded at 3×10^5^ cells per well in 6 well plates for RNA and protein experiments. Two 10 cm plates seeded at a density of 3.5×10^6^ cells were pooled for each ChIP replicate.

### Estradiol Treatment

For hormonal deprivation, cells were cultured in phenol-red free media supplemented with 10% charcoal dextran-treated fetal bovine serum for 72 hours. Cells were treated with 100 nM Estradiol (Sigma) for 45 minutes for ChIP experiments, 6 hours for RNA experiments or 24 hours for the luciferase reporter assay.

### Chemicals and siRNAs

17-β-estradiol (Sigma), Lipofectamine RNAiMAX (Invitrogen), Genejuice (Merck), RAD21 siRNA (h): J006822-06 (Dharmacon), ESR1 siRNA (h): HSS103377 (Invitrogen), Negative Control siRNA medium GC (Invitrogen).

### Western Blotting

Cells were lysed in Radio-Immunoprecipitation Assay (RIPA) buffer containing protease inhibitors (Complete™, Roche) followed by freeze-thaw. Protein concentration was measured using Bicinchoninic Acid (BCA) assay (Pierce, Thermoscientific) and equal amounts of protein were separated by electrophoresis of 10% polyacrylamide gels. For enhanced chemiluminescent (ECL) detection protein was transferred onto PVDF membranes (Thermoscientific). Membranes were blocked with 10% skim milk and then probed with overnight at 4°C with a rabbit anti-RAD21 antibody (1∶1000, Ab992, Abcam) or rabbit anti-ERα antibody (1∶2000, HC20, Santa Cruz) diluted in 5% milk, Tris Buffered Saline (TBS) and 0.1% Tween 20. The membranes were washed with TBS-Tween and incubated with Horseradish-Peroxidase (HRP) conjugated secondary antibody (1∶2000, Sigma) for 1 hour at room temperature. Detection was performed using RapidStep™ ECL as described by the manufacturer (Calbiochem). The Odyssey® Infrared detection system was also used for Western visualization. Proteins were transferred to Nitrocellulose membranes (Thermoscientific) and blocked using the Odyssey® Casein Block and Antibody Diluent (LiCor), according to the manufacturer’s instructions. The membranes were incubated with Rabbit anti-RAD21 (1∶1000, Ab992, Abcam) and mouse anti-γ-actin (1∶500, Sigma) diluted in Odyssey® Casein Block and Antibody Diluent with 0.1% Tween-20 at 4°C overnight. After washing with TBS-Tween, the membranes were incubated with IRDye®-conjugated antibodies (1∶15,000, LiCor) for one hour at room temperature, and visualized with the Odyssey® CLx Infrared imaging system (LiCor).

### Gene Expression Analysis

Total RNA was isolated from treated cells using Trizol Reagent (Invitrogen), according to the manufacturer’s instructions. RNA was processed using the Qiagen RNeasy mini kit with DNAse treatment (Qiagen). cDNA was transcribed using the Superscript III first strand synthesis system, according to the manufacturer’s instructions (Invitrogen). Real-time quantitative PCR was performed using Platinum® SYBR® Green qPCR SuperMix-UDG with ROX (Invitrogen) on an ABI 7300 Real Time PCR System (Applied Biosystems). qBase Plus (Biogazelle) was used to quantify the transcript levels of each gene, relative to endogenous controls for each sample. For experiments using MCF7 cells, *GAPDH*, *RPL13a* were used as housekeeping genes. For experiments using T47D or MDA-MB-231 cells *Cyclophilin* was used in addition to *GAPDH* and *RPL13a*. Results are represented as fold change in expression level relative to control sample (mean ± SEM, n  = 3). Primer sequences can be found in Table S1.

### Chromatin Immunoprecipitation

For each sample, 1×10^7^ cells were treated with estradiol or vehicle for 45 minutes prior to fixation with 11% formaldehyde for ten minutes. The fixing solution was quenched with glycine. ChIP was performed according to the method described by Vaisanen *et al* [109]. Briefly, cells were lysed in a sodium dodecyl sulfate (SDS)-lysis buffer containing protease inhibitors prior to sonication (Vibra Cell VCX130 Sonicator, Sonics). Soluble chromatin was measured by nanodrop. Equal amounts of diluted chromatin were pre-cleared with ssDNA/protein A agarose beads (Millipore). IPs were performed, using RAD21 and ERα antibodies (as outlined in Western Blotting), with rotation at 4° overnight. Immunocomplexes were collected using ssDNA/protein A agarose beads and washed sequentially with low salt, high salt, Lithium Chloride and Tris-EDTA (TE) buffers. Immunocomplexes were eluted using SDS and NaHCO_3_ and treated with RNAse A and Proteinase K. DNA was recovered using Phenol:Chloroform:Isoamyl alcohol (Invitrogen) and precipitated with ethanol. Immunoprecipitation was quantitated using qPCR (see above). Binding was calculated relative to a pre-cleared input sample and a no antibody control, and normalized to a site where no binding was observed (NEG). Data shown are the mean (+/− SEM) of at least 3 independent IPs. Primer sequences can be found in Table S1.

### Luciferase Assay

24 hours before transfection MCF7 cells were washed and treated with 10 nM ICI 182780 (Sigma) to inhibit estrogen activity. Cells were transfected with 10 nM siRNA using Lipofectamine RNAiMAX (Invitrogen) according to the manufacturer’s instructions. The cells were seeded into a flat-bottomed white 96 well plate (Nunc) at a density of 10,000 cells per well. Six hours after siRNA transfection, 100 ng of ERE-Reporter, Negative Control or Positive Control plasmids from the ERE Cignal Reporter Assay Kit (Qiagen) were transiently transfected using GeneJuice (Merck). Cells were treated with either vehicle (ethanol) or 100 nM 17-β-Estradiol, 48 hours after siRNA transfection. The Dual-Glo® Luciferase Assay System (Promega) was used according to the manufacturer’s instructions to measure activation of the reporter plasmid 24 hours after estradiol treatment. Values were normalized to *Renilla* luciferase activity; data are presented as relative luciferase values (mean ± SEM, n  = 5). Transfection efficiency was measured by counting cells expressing green fluorescent protein (GFP) from a co-transfected GFP plasmid, and was similar between all treatments.

### Statistical Analysis

GraphPad Prism software was used for graphing and statistical analysis. Q-PCR data were analyzed using one-way ANOVA with post-Tukey’s multiple comparison test with a significance level set at p<0.05. ChIP data were analyzed by two-way ANOVA with Bonferroni post-test with a significance level set at p<0.05.

## Supporting Information

Figure S1 RNA Polymerase II (Pol II) binding is enriched at the P2 promoter of transcriptionally active *MYC.* Pol II binding in estradiol treated MCF7 cells and in MDA-MB-231 cells was analyzed by ChIP. The data shown is percent of chromatin input. The bar graph represents the mean +/− SEM of three independent experiments. Pol II predominantly binds the promoter region in MCF7 cells, however in MDA-MB-231 cells Pol II also binds to a site 0.8 kb upstream of the transcriptional start site. ChIP primer sequences are listed in Table S1. A scale diagram of primer positions relative to the *MYC* gene and promoters is shown in Figure S3.
(PDF)

Figure S2 Depletion of RAD21 protein is accompanied by a reduction in RAD21 chromatin binding. MCF7 cells were transfected with Control or RAD21 siRNA (10 nM) for 48 hours and then fixed following treatment with vehicle (V) or 100 nM estradiol (E) for 45 minutes. RAD21 binding was analyzed using ChIP. Data shown is fold enrichment; binding was calculated relative to input chromatin and normalized against the NEG site where no binding was observed. The bar graph shows the mean +/− SEM of three independent experiments. The * and **** symbols indicate a significant (p<0.05 and p<0.001 respectively) reduction in RAD21 binding between estradiol treated Control siRNA and RAD21 siRNA transfected MCF7 cells. ChIP primer sequences are listed in Table S1. A scale diagram of primer positions relative to the *MYC* gene and promoters is shown in Figure S3.
(PDF)

Figure S3 Scale diagram of locations of ChIP primers in relation to *MYC* gene and promoters.
(PDF)

Table S1 Primer sequences and chromosomal locations for Chromatin Immunoprecipitation, and qPCR primer sequences.
(XLSX)
