# Supplementary figures and images for: Cohesin Is Required for Activation of MYC by Estradiol
Source: PLoS One. 2012 Nov 8;7(11):e49160. doi: 10.1371/journal.pone.0049160 (PMC3493498; doi:10.1371/journal.pone.0049160)

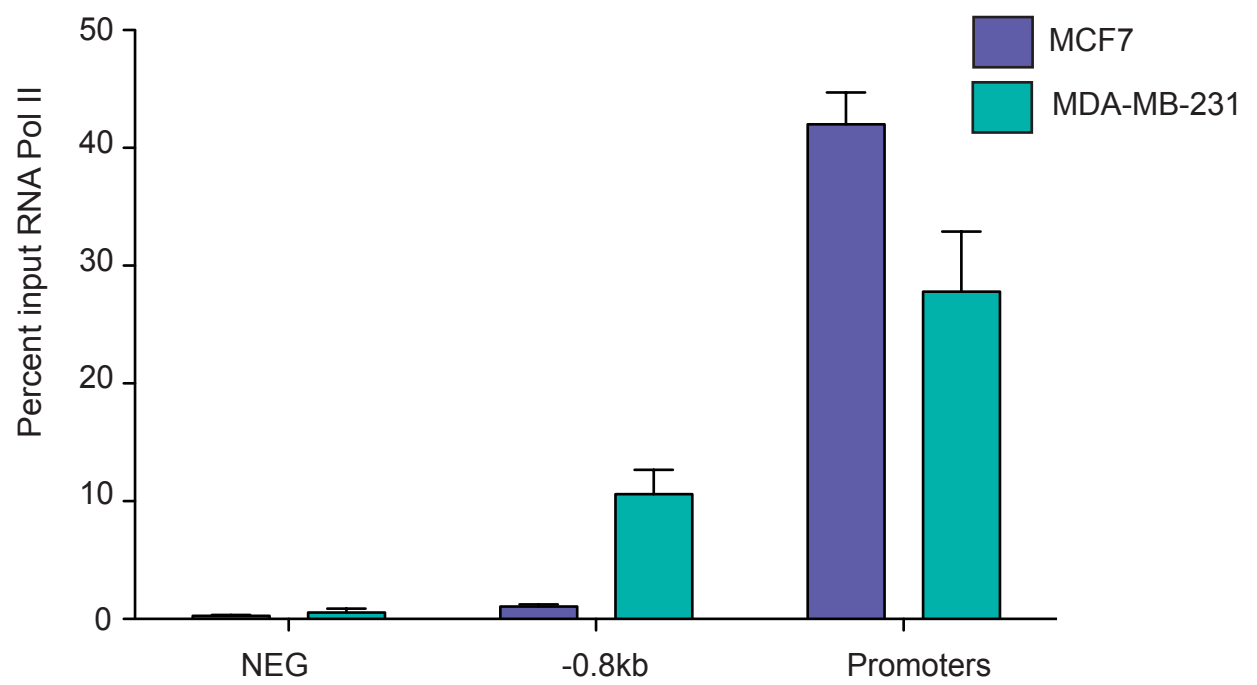

Supplement: Figure S1 — RNA Polymerase II (Pol II) binding is enriched at the P2 promoter of transcriptionally active MYC. Pol II binding in estradiol treated MCF7 cells and in MDA-MB-231 cells was analyzed by ChIP. The data shown is percent of chromatin input. The bar graph represents the mean +/− SEM of three independent experiments. Pol II predominantly binds the promoter region in MCF7 cells, however in MDA-MB-231 cells Pol II also binds to a site 0.8 kb upstream of the transcriptional start site. ChIP primer sequences are listed in Table S1. A scale diagram of primer positions relative to the MYC gene and promoters is shown in Figure S3. (PDF) [file pone.0049160.s001.pdf]

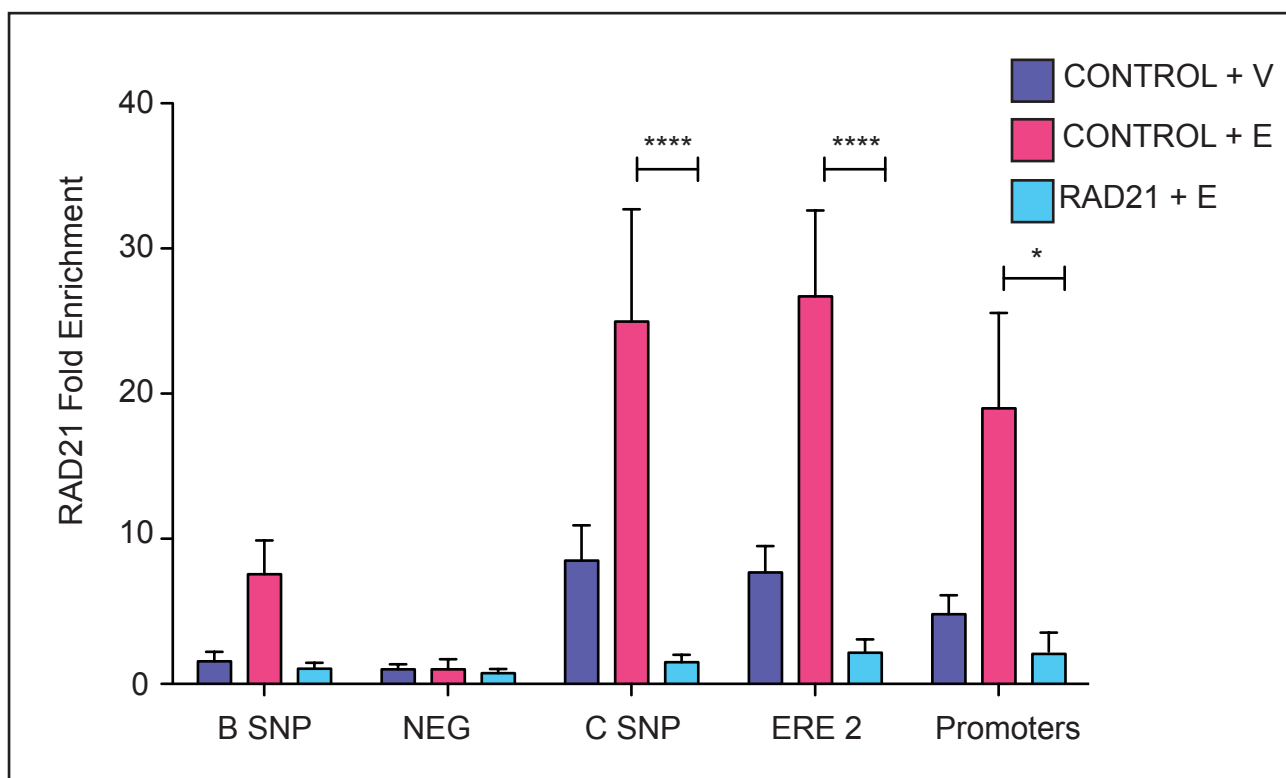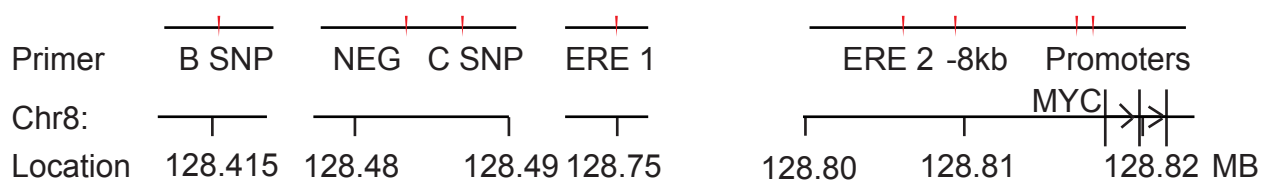

Supplement: Figure S2 — Depletion of RAD21 protein is accompanied by a reduction in RAD21 chromatin binding. MCF7 cells were transfected with Control or RAD21 siRNA (10 nM) for 48 hours and then fixed following treatment with vehicle (V) or 100 nM estradiol (E) for 45 minutes. RAD21 binding was analyzed using ChIP. Data shown is fold enrichment; binding was calculated relative to input chromatin and normalized against the NEG site where no binding was observed. The bar graph shows the mean +/− SEM of three independent experiments. The * and **** symbols indicate a significant (p<0.05 and p<0.001 respectively) reduction in RAD21 binding between estradiol treated Control siRNA and RAD21 siRNA transfected MCF7 cells. ChIP primer sequences are listed in Table S1. A scale diagram of primer positions relative to the MYC gene and promoters is shown in Figure S3. (PDF) [file pone.0049160.s002.pdf]

Location on  
Chromosome 8:  
(Hg16)

128,805,668

128,809,668

128,813,668

128,817,668

128,821,668

ChIP primers

ERE 2

-8 kb

-0.8 kb

Promoters

*MYC*

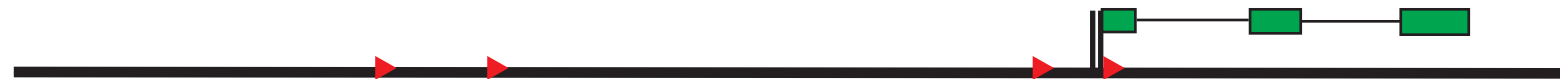

Supplement: Figure S3 — Scale diagram of locations of ChIP primers in relation to MYC gene and promoters. (PDF) [file pone.0049160.s003.pdf]
